# Dezocine is a Biased Ligand without Significant Beta-Arrestin Activation of the mu Opioid Receptor

**Published:** 2022-03-01

**Authors:** John Grothusen, Wenzhen Lin, Jin Xi, Giulia Zanni, Gordon A. Barr, Renyu Liu

**Affiliations:** 1 Department of Anesthesiology and Critical Care, Perelman School of Medicine at the University of Pennsylvania, USA; 2 Department of Biochemistry and Molecular Biology, School of Preclinical Medicine, Guangxi Medical University, China; 3 Department of Anesthesiology and Critical Care Medicine, Children’s Hospital of Philadelphia, USA; 4 Department of Psychiatry, Columbia University, USA

## Abstract

Dezocine is an opioid that was used in clinical practice for acute pain management in the US (1986 to 2011) and is currently in use in China. It is not listed as a controlled substance in the US due to no reported cases of addiction. Dezocine is a partial agonist at the mu opioid receptor (MOR); however, it is unclear whether dezocine can activate both the G protein pathway and the beta-arrestin pathway. In this study we hypothesized that dezocine does not activate the beta-arrestin pathway, which could be the potential molecular mechanism by which dezocine is not addictive or at least less addictive than other classic opioids. Both morphine, a MOR full agonist and buprenorphine, a partial MOR agonist similar to dezocine, were used for comparison purposes. The major side effects of dezocine in clinical usage are its gastrointestinal side effects and first pass effects; therefore, we explored the possibility of administering dezocine intranasally in rodents to demonstrate the feasibility of intranasal administration for new clinical usage purposes. With proper formulation it is possible to administer dezocine intranasally to achieve a high concentration in the brain in the rodent model. The results indicate that dezocine does not activate the beta-arrestin pathway in MOR. Intranasal delivery of dezocine achieves a much higher medication concentration in the blood and brain as compared to intraperitoneal injection. It also persists a longer time before it falls below detection in the blood. This study provides a possible explanation of why dezocine is not addictive or at least less addictive than other commonly used opioids. This study also demonstrates that intranasal administration offers an alternative strategy for its potential clinical applications.

## Introduction

Dezocine is an opioid, distinct from classic potent opioids such as morphine, since it is a mixed partial mu opioid receptor (MOR) agonist and a kappa opioid receptor (KOR) antagonist that does not show strong addictive side effects. Dezocine also inhibits the norepinephrine (NET) and serotonin transporters (SERT), which are related to pain and depression pathways [1]. Dezocine is not categorized as a controlled substance in the US despite its designation as an opioid. Dezocine had been used for post-operative pain treatment for over two decades in the USA, and currently is used in China for the perioperative period [2,3]. There are no known reports of dezocine addiction in the English literature. Dezocine is equipotent with morphine for post-operative pain, and is faster acting with fewer adverse effects than morphine [3,4]. Dezocine has been gaining in use in China for cancer pain [5], and there is evidence that dezocine may also be effective for treating neuropathic pain [6]. It is reported that the side effects of opioids are related to their activation on the beta-arrestin pathway [7]. It has been suspected that dezocine could potentially be a biased ligand for MOR without significant activation of the beta-arrestin pathway [8]. Therefore, in this study, we hypothesized that dezocine would have no significant activation of the MOR beta-arrestin pathway. To expand the clinical usage of dezocine, we also explored whether dezocine could be delivered intranasally to achieve therapeutic concentrations in the blood and brain using a rodent model.

## Methods

All the chemicals used in this study were commercially obtained and were either analytical or pharmaceutical grade with more than 98% purity. Nano-dezocine (dezocine complexed with 2-hydroxypropyl-γ-cyclodextrin) is a patented formulation produced in our laboratory [9]. HTLA cells, an HEK293 cell line stably expressing a tTA-dependent luciferase reporter and a beta-arrestin-TEV fusion gene, were kindly provided by the laboratory of Bryan L. Roth MD, PhD at the University of North Carolina at Chapel Hill [10]. The animal protocols were approved by the Institutional Animal Care and Use Committees at the University of Pennsylvania or Children’s Hospital of Philadelphia.

### Beta-arrestin activation assay

The beta-arrestin recruitment study was performed in an HTLA cell line stably transfected with the human mu opioid receptor as previously described [11]. Activation of beta-arrestin signaling was quantified by a Tango assay using the HTLA cells [12]. HTLA cells are a cell line that is stably transfected with beta-arrestin coupled with a TEV protease that releases a promotor to initiate transcription and translation of luciferase by the cells when ligand binding to MOR causes activation of the beta-arrestin [12]. HTLA cells were thawed and plated overnight, transfected the following day with a plasmid coding for MOR receptor, trypsinized and plated on 96 well plates. The adherent cells were exposed to ligands (dezocine, morphine or buprenorphine) overnight. The following day media was removed from the wells and replaced with Bright-Glo Luciferase Assay System reagent (Promega). Luminescence generated from the luciferase enzyme which was produced by HTLA cells with beta-arrestin recruitment which then reacts with substrate in the Bright-Glo reagent and was read on a Synergy H1 microplate reader (BioTek).

### Dezocine blood levels following intraperitoneal and intranasal dosing

Adult male and female Sprague Dawley (Taconic Biosciences) rats (n = 3 of each sex for each route) were used. Nano-dezocine [9] was administered either intraperitoneally (IP) (2.0 mg/kg in a volume of 1 ml/kg) or intranasally (IN) (2.0 mg/kg in a maximum volume of 75 μl/kg total for both nostrils). At 15, 30, 60, 120, 240 and 480 minutes after administration, rats were lightly anesthetized with isoflurane and blood was drawn (approximately 200 μl) from the ventral tail vein. Blood was placed into heparinized tubes, centrifuged, and plasma was collected and frozen. Dezocine was measured by Liquid Chromatography-Mass Spectroscopy (LC-MS) using a Thermo Scientific Q-Exactive HF-X mass spectrometer coupled to the Thermo Scientific Vanquish Horizon UHPLC system. Dezocine was quantified using the full MS data by extracted ion chromatogram (XIC) of 246.1852 m/z at 5 ppm mass tolerance and the accurate retention time. Quantitative recovery of dezocine was obtained using ice cold 80% methanol as the extraction solvent in experimental plasma samples.

### Dezocine blood and brain levels following intranasal dosing

Nano-dezocine was administered by the intranasal route to a separate cohort of adult Sprague-Dawley rats. A maximum of ten microliters of nano-dezocine (24 mg/mL) was administered by micropipette into each nostril (total 20 μL) for a final dose of 2 mg/kg. Rats were sacrificed at 30 and 60-minutes post dosing, and following decapitation, trunk blood and brains were rapidly collected. Blood samples were collected into heparinized tubes and after centrifugation, plasma was collected and frozen. Brains were rapidly removed and dropped directly into liquid nitrogen and stored at −80 °C. Plasma was thawed and extracted with ice cold 80% MeOH and centrifuged. The supernatants were analyzed for dezocine content using HPLC-MS. Brain samples were weighed, homogenized in ice cold 80% MeOH, and following centrifugation the supernatants were analyzed for dezocine content using LC-MS as described above.

### Statistical analysis

Data are either presented as mean ± SE or SD (standard error or standard deviation) as indicated in each figure. The statistical analysis and graph generation were performed using GraphPad Prism (version 9.2.0, GraphPad SoftwareSan Diego, CA). Either 2-way analysis of variance or unpaired tests were used for statistical analysis as needed. Details of the statistical analyses are presented in each figure legend with P less than 0.05 considered statistically significant.

## Results

### Dezocine does not activate beta-arrestin pathway in human MOR

**Figure 1:**
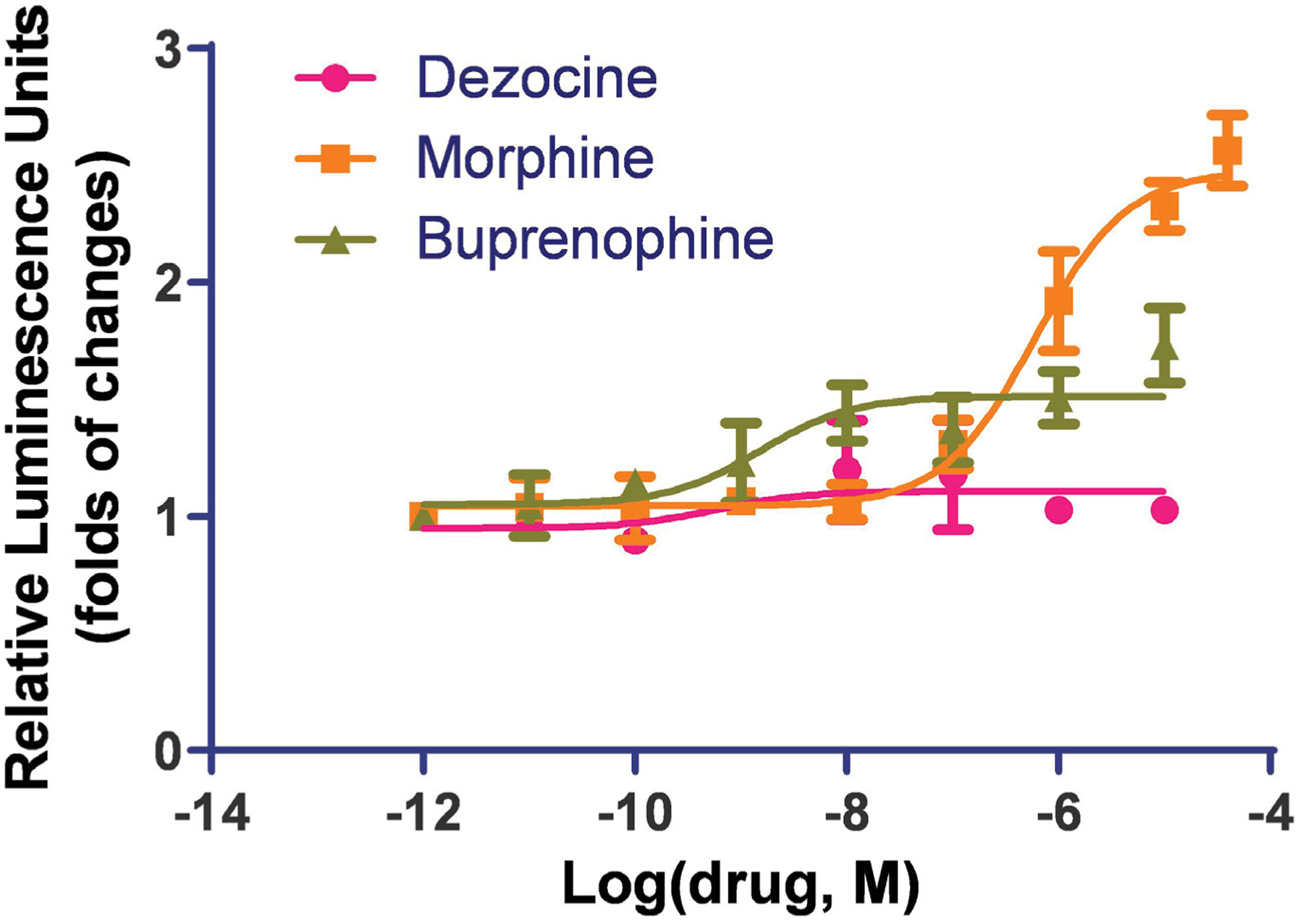
Dezocine does not activate the beta-arrestin pathway in human MOR. The beta-arrestin recruitment study was performed in an HTLA cell line stably transfected with the human mu opioid receptor. Both morphine and buprenorphine induced beta-arrestin recruitment. Buprenorphine indicated partial activation and morphine indicated full activation of the MOR in the beta-arrestin pathway. Dezocine did not induce beta-arrestin recruitment. Data are presented as mean ± standard error.

Both morphine and buprenorphine induced beta-arrestin recruitment. Buprenorphine indicated partial activation with an EC50 of 1.5 ± 0.07 nM, and morphine indicated full activation of the MOR in the beta-arrestin pathway with an EC50 of 600 ± 0.07 nM. Dezocine did not induce such activities (Figure 1). Buprenorphine can activate beta-arrestin recruitment at a much lower concentration than that for morphine, however it did not reach a full activation as observed for morphine.

### Intraperitoneal versus intranasal administration of nano-dezocine

**Figure 2:**
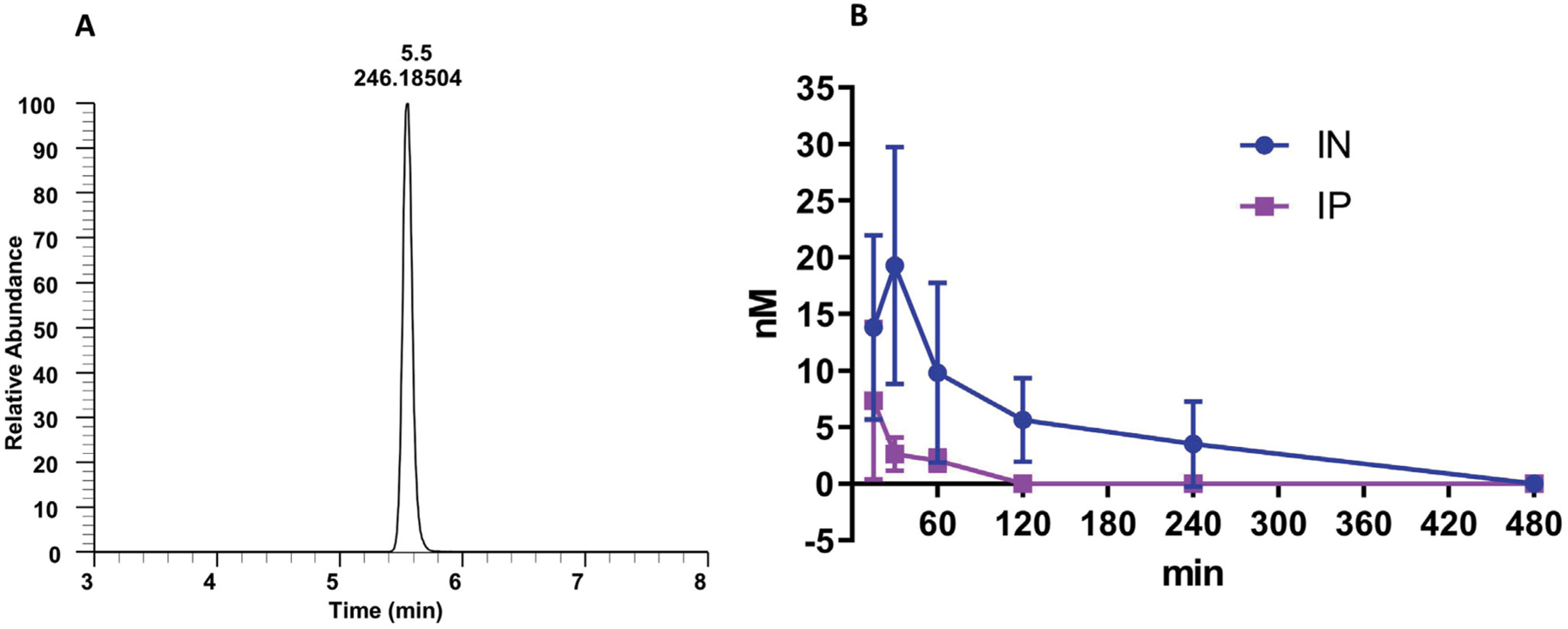
Kinetics of nano-dezocine (A) single peak of dezocine in a blood sample; (B) Comparison of dezocine concentration in plasma with intraperitoneal (IP) and intranasal (IN) dosing (2.0 mg/kg) in adult male and female rats. Data are presented as mean ± standard deviation. Significant difference between the two groups was observed using 2-way Analysis of variance to compare the difference between the two groups with a p value of 0.002. n = 3 per route of administration per sex.

The intranasal route resulted in much higher blood levels and a longer duration than intraperitoneal injection (Figure 2). Intraperitoneal administration of nano-dezocine led to a rapid rise of plasma dezocine concentrations at 15 minutes, followed by a very rapid decrease in plasma concentration, likely due to significant first-pass metabolism. However, plasma concentrations of intranasal nano-dezocine continued to increase for an additional 15 minutes longer than the IP dosing before declining more slowly.

### Brain and plasma levels with intranasal administration of nano-dezocine

**Figure 3:**
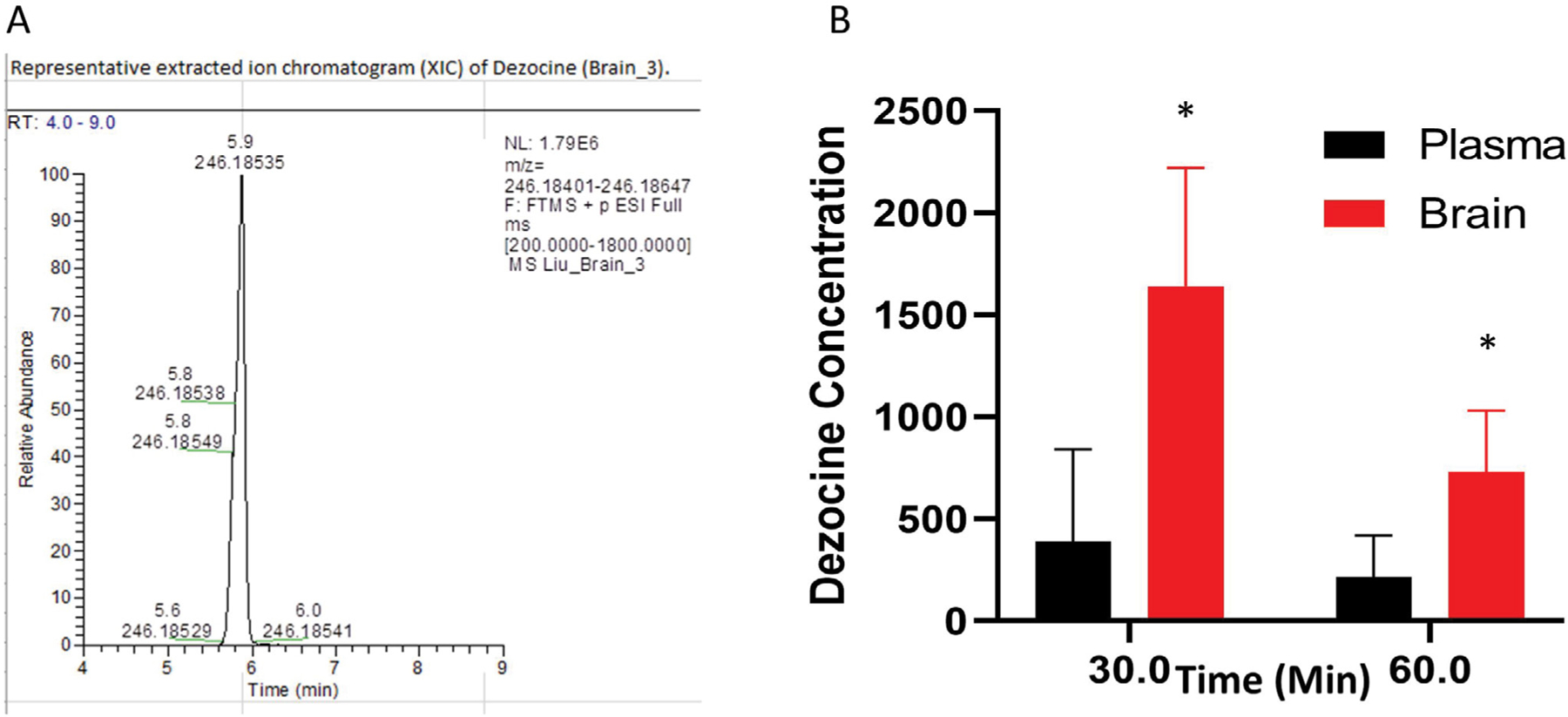
Dezocine in brain and plasma (A) Representative extract ion chromatogram of dezocine in the brain; (B) The content of dezocine (pMol/gm) in brain and plasma (pMol/gm) following intranasal administration. In each time point, the statistical analysis was performed using an unpaired test. p = 0.027 for the point at 30 min; p = 0.028 for the point at 60 min, *indicates statistically significant as compared to the concentration in the plasma. Data are presented as mean ± standard deviation (n = 4 per time point).

The intranasal route resulted in much higher concentrations of dezocine in the brain than in the plasma (Figure 3). Moreover, this elevated concentration following intranasal administration persisted to at least 60 minutes post-treatment.

## Discussion

The results of this study provide evidence that dezocine is a biased opioid on MOR without significant activation on the beta-arrestin pathway. This could potentially be one of the mechanisms as to why dezocine, a drug with a long history of use in the US and China for pain management, is without strong evidence of addiction. Beta-arrestin activation has been considered as the key molecular mechanism of adverse effects of opioid analgesics including addiction [7,13].

The most notorious side effect of opioids is addiction, which is the mainstay of the current opioid crisis. To develop receptor signaling biased opioids has been one of the major efforts in drug development for pain killers without addiction properties [10,14]. The discovery that dezocine is a G protein biased opioid may help its expansion or its resurrection in clinical usage as recently discussed [8]. It is advisable that when an opioid medication is needed or indicated for moderate and severe pain control, it is better to start with a non-addictive or less addictive opioid rather than starting with strong addictive pain medications. This could help to solve the current problem of the opioid crisis. Also, dezocine is finding use for cancer pain management in China due to its analgesic properties [5] as well as its potential tumor inhibition properties [15,16]. Dezocine has potential therapeutic value for neuropathic pain, without evidence of tolerance or dependence, as demonstrated in a rodent model of neuropathic pain [6].

It was demonstrated that a G protein biased ligand at the MOR has fewer gastric and respiratory depression side effects [17]. Although dezocine can induce respiratory depression, the respiratory depressant effect of dezocine is subject to a ceiling effect due to its known partial MOR agonist properties. The discovery in this study indicating its inactivity on the beta-arrestin pathway might be another molecular mechanism related to its lesser respiratory depression effects. There were no reports of dezocine overdose related deaths found using Pubmed as searched on January 28^th^, 2022 using the keyword of “dezocine overdose death”. Maximal respiratory depression occurs at a total cumulative dose of about 0.43 milligrams per kilogram of body weight (mg/kg) without further respiratory depression occurring even increasing the dose an additional 0.14 mg/kg in healthy human subjects [18,19]. Dezocine induced respiratory depression can be rescued by naloxone completely [19]. Although buprenorphine also has ceiling effects on respiratory depression, the respiratory depression from buprenorphine can be induced as low as 0.003 mg/kg, a dose 140 times lower than the dose causing respiratory depression from dezocine in healthy human subjects. Such respiratory depression from buprenorphine is not always reliably rescued with naloxone [20]. Deaths related to buprenorphine overdose have been reported [21].

Dezocine has been found to inhibit both the serotonin and norepinephrine uptake transporters [1]. This led to the investigation and discovery that dezocine has antidepressant activity in patients treated post-operatively for pain [22]. Anti-depressant activity of dezocine was also verified in a rodent model [23]. It has been known that depression is a major comorbidity in chronic pain and opioid use disorder [24]. Substituting dezocine for methadone or buprenorphine for opioid use disorder would have the added benefit of treating the major comorbidity depression along with its other favorable properties of reduced respiratory depression and lack of addiction.

A hurdle in the expanded use of dezocine has been the problem of formulation. Solubility issues made dezocine available only as an acidic solution which may cause injection pain. First pass metabolism issues limited development of oral formulations. Here we demonstrated that the intranasal route, using our patented nano-dezocine formulation [9] is a viable and potentially clinically useful alternative route of administration. This route would be a sound alternative for outpatient treatment of acute pain, withdrawal syndrome and addiction. The results indicated that dezocine can achieve much higher and longer time of the detectable plasma concentration with the intranasal route, indicating a longer time of therapeutic levels. Detailed pharmacokinetics need to be evaluated in the future when such strategy is transitioned for clinical applications.

In conclusion, dezocine is a unique partial MOR agonist without significant beta-arrestin recruitment properties. Due to such unique molecular pharmacological properties, its clinical usage and value needs to be revisited and re-evaluated. Intranasal administration could potentially be an alternative strategy for expanded clinical indications.
